# Genetic changes found in a distinct clade of Enterovirus D68 associated with paralysis during the 2014 outbreak

**DOI:** 10.1093/ve/vew015

**Published:** 2016-06-15

**Authors:** Yun Zhang, Jing Cao, Song Zhang, Alexandra J. Lee, Guangyu Sun, Christopher N. Larsen, Hongtao Zhao, Zhiping Gu, Sherry He, Edward B. Klem, Richard H. Scheuermann

**Affiliations:** 1 J. Craig Venter Institute, La Jolla, CA, USA; 2 Department of Statistical Science, Southern Methodist University, Dallas, TX, USA; 3 Department of Clinical Science, University of Texas Southwestern Medical Center, Dallas, TX, USA; 4 Vecna Technologies, Greenbelt, MD, USA; 5 Northrop Grumman Health Solutions, Rockville, MD, USA; 6 Department of Pathology, University of California, San Diego, La Jolla, CA, USA

**Keywords:** Enterovirus D68, EV-D68, comparative genomics, genotype–phenotype correlation, evolution, phylogenetics, Virus Pathogen Resource (ViPR), meta-CATS, poliovirus

## Abstract

Enterovirus D68 (EV-D68) caused a severe respiratory illness outbreak in the United States in 2014. Reports of acute flaccid myelitis (AFM)/paralysis (AFP) in several independent epidemiological clusters of children with detectable EV-D68 have raised concerns that genetic changes in EV-D68 could be causing increased disease severity and neurological symptoms. To explore the potential link between EV-D68 genetic variations and symptom changes, we performed a series of comparative genomic analyses of EV-D68 2014 outbreak isolate sequences using data and analytical tools in the Virus Pathogen Resource (ViPR; www.viprbrc.org). Our results suggest that (1) three distinct lineages of EV-D68 were co-circulating in 2013 and 2014; (2) isolates associated with AFM/AFP belong to a single phylogenetic subclade – B1; (3) the majority of isolates from the B1 subclade have 21 unique substitutions that distinguish them from other isolates, including amino acid substitutions in the VP1, VP2, and VP3 capsid proteins and the 3D RNA-dependent RNA polymerase, and nucleotide substitutions in the internal ribosome entry sequence (IRES); (4) at 12 of these positions, B1 isolates carry the same residues observed at equivalent positions in paralysis-causing enteroviruses, including poliovirus, EV-D70 and EV-A71. Based on these results, we hypothesize that unique B1 substitutions may be responsible for the apparent increased incidence of neuropathology associated with the 2014 outbreak.

## 1. Introduction

The *Picornaviridae* family is composed of non-enveloped, single-stranded, positive-sense RNA viruses. The genomes of picornaviruses encode a single open-reading frame that is translated into a long polyprotein, which is subsequently processed into mature peptides by virus-encoded proteases (Racaniello 2006; ViralZone 2015). The *Enterovirus* genus of *Picornaviridae* consists of many important human viral pathogens, including human rhinoviruses (HRV), as the most common viral agents of the common cold, polioviruses (PV-1, PV-2, and PV-3), causing poliomyelitis within the *Enterovirus C* species, enterovirus A71 (EV-A71), causing a variety of neurological diseases, and enterovirus D68 (EV-D68) (Racaniello 2006). Before the summer of 2014, EV-D68 had been typically associated with respiratory illnesses, with only two isolated cases associated with neurologic symptoms (Khetsuriani et al. 2006; Kreuter 2011). From August 2014 to January 2015, EV-D68 caused a severe respiratory illness outbreak in the United States, with 1,153 confirmed EV-D68 cases in 49 U.S. states and the District of Columbia (CDC 2015a). This outbreak coincided with a nationwide spike in polio-like illnesses, with 115 cases in 34 states meeting the Acute Flaccid Myelitis (AFM) definition (CDC 2016). Statistical analyses of the AFM cases in Colorado (Messacar et al. 2015) and California (Van Haren et al. 2015) both suggested that the number of AFM cases was significantly higher during the EV-D68 outbreak in comparison with historical controls. Among these AFM cases, several independent epidemiological clusters of children tested positive for EV-D68 (Ayscue et al. 2014; Greninger et al. 2015; Messacar et al. 2015). For example, a cluster of EV-D68 positive AFM cases was reported at the Children’s Hospital Colorado (Messacar et al. 2015), and seven AFM-associated EV-D68 illness cases were reported in California with no spatial clustering noted (Van Haren et al. 2015). Outside the U.S., Canada (Skowronski et al. 2015), France (Lang et al. 2014), Norway (Bragstad et al. 2015), and Australia (Levy et al. 2015), each reported a small number of EV-D68 positive AFM cases. These reports have raised concerns that the EV-D68 viruses associated with this recent outbreak could be causing increased disease severity and neurological symptoms.

To explore the evolutionary source of the EV-D68 outbreak viruses and the potential link between a possible novel lineage and the apparent changes in symptomatology associated with the 2014 EV-D68 outbreak, we performed a series of comparative genomics analyses of the EV-D68 2014 outbreak isolates to identify amino acid and nucleotide substitutions in 2014 outbreak isolates that could be contributing to increased disease severity and neurological symptoms.

## 2. Materials and methods

### 2.1 Sequence retrieval

All sequences were retrieved from the Virus Pathogen Resource (ViPR) (Pickett et al. 2012) – *Picornaviridae* family (http://www.viprbrc.org/brc/home.spg?decorator=picorna) on 9 February 2016. Sequences from the same genomic regions were aligned using the MUSCLE algorithm (Edgar 2004) implemented in ViPR for sequence quality assessment. Truncated (VP1 only) and questionable sequences were removed from the datasets. ViPR generates predicted mature peptide sequences for all enterovirus isolate sequences as a consistent source of mature peptide sequence annotations that were used for all protein-based analyses. The remaining EV-D68 sequences after these quality control steps were used for downstream analyses.

To compare EV-D68 sequences with those of other enteroviruses that are associated with neurological illnesses, the full-length EV-D70 isolate (J670/71), two AFP-associated EV-A71 isolates (AFP2001064/EV71/GX/CHN/2001 and AFP2001071/EV71/GX/CHN/2001), and representative neurovirulent poliovirus isolates (PV-1: Human poliovirus 1 Mahoney; PV-2: PV2/Bel-2; PV-3: P3/Leon/37) were used.

### 2.2 Phylogenetic analysis

Phylogenetic relationships between the nucleotide sequences of complete VP1 coding regions were inferred using RAxML (Stamatakis et al. 2005) with 1,000 bootstrap runs on the ViPR site. The resulting phylogenetic tree was subsequently viewed in Archaeopteryx on ViPR for decoration based on year of isolation.

### 2.3 Phylogeny–phenotype association analysis

Each EV-D68 isolate was assigned a neurovirulence trait for phylogeny–phenotype association analysis. Isolates associated with either AFM/AFP or encephalitis case reports were classified as neurovirulent. EV-D68 isolates that were not explicitly associated with an AFM/AFP or encephalitis case report were classified as non-neurovirulent, based on the fact that historically EV-D68 had been predominantly linked to respiratory symptoms only, with the exception of two cases for which no genome sequence data is available. In order to determine if the neurovirulent isolates demonstrated a non-random distribution during viral evolution, the same VP1 nucleotide dataset used in phylogenetic analysis was first input into the Bayesian Evolutionary Analysis Utility (BEAUTi) and then the Bayesian Evolutionary Analysis Sampling Trees (BEAST). The BEAST tree was subsequently input into the Bayesian Tip-association Significance (BaTS) program (Parker et al. 2008) to calculate parsimony score (PS), association index (AI), and monophyletic clade (MC) statistics. The PS statistic calculates the number of gains/losses of a trait under investigation in the parsimony phylogeny, with low PS scores indicating strong phylogeny-trait association. The AI score measures the trait frequency of descendent sequences at each bifurcating node, thus assessing imbalance in the phylogeny. Low AI values indicate strong phylogeny–trait associations. MC reports the size of the largest monophyletic clade whose tips all share the same trait under investigation, and is positively correlated with the phylogeny–trait association. Whether the resulting scores are statistically significant is determined by comparing the observed scores with an empirical null distribution for that statistic generated by a randomization bootstrap approach.

### 2.4 Comparison of B1 and non-B1 EV-D68 isolates and related enterovirus sequences

Genetic differences between EV-D68 B1 and non-B1 isolates were identified by two separate statistical tests, using protein sequences for mature peptides and nucleotide sequences for 5’UTR. The first statistical test is a Chi-squared statistical test implemented in the ViPR meta-CATS tool (Pickett et al. 2013). The second comparison statistical test accounts for isolate relatedness due to shared phylogenetic ancestry (see Supplementary method). In short, the conventional two-proportion *z*-test to determine whether the difference between two proportions is significant assumes that the samples in each group are independent. The second statistical test used in this study extends the *z*-test by allowing the samples (i.e. isolates) to be dependent. For each position, the test statistic is constructed based on the comparison of mutation proportions between the B1 and non-B1 isolates. The approach assumes a common evolutionary correlation matrix among the isolates across all positions, where the correlation matrix is a Bayesian estimate based on the sample correlation matrix. By incorporating the correlation matrix in the test statistic, the test can account for the dependency among the isolates due to shared evolutionary relatedness.

For each significant position identified by these statistical tests, sensitivity and specificity were calculated based on the following formulae:

(1) $$Sensitivity=\frac{TP}{TP+FN}$$

(2) $$Specificity=\frac{TN}{TN+FP}$$

where *TP* is the number of isolates containing the B1-representative residue/nucleotide in the subclade B1 group, *FN* is the number of isolates containing non-representative residue(s)/nucleotide(s) in the B1 group, *TN* is the number of isolates containing non-representative residue(s)/nucleotide(s) in the non-B1 group, and *FP* is the number of isolates containing the B1-representative residue/nucleotide in the non-B1 group.

Positions with a Chi-squared *P* value < 0.05, *P* value corrected for evolutionary relatedness among isolates < 0.05, specificity > 0.80, and sensitivity > 0.75 in the subclade B1 group were considered to be significant.

To compare the B1 isolates with paralysis-causing poliovirus, EV-D70, and EV-A71 enteroviruses, EV-D68 sequences were aligned with the corresponding sequences of representative poliovirus, EV-D70, and EV-A71 viruses using the MUSCLE algorithm (Edgar 2004) on the ViPR site, and the statistically significant EV-D68 positions were mapped to the equivalent positions in poliovirus, EV-D70, and EV-A71 viruses.

## 3. Results

### 3.1 Phylogenetic analysis suggests three distinct clades co-circulating in 2013 and 2014

**Figure 1.**
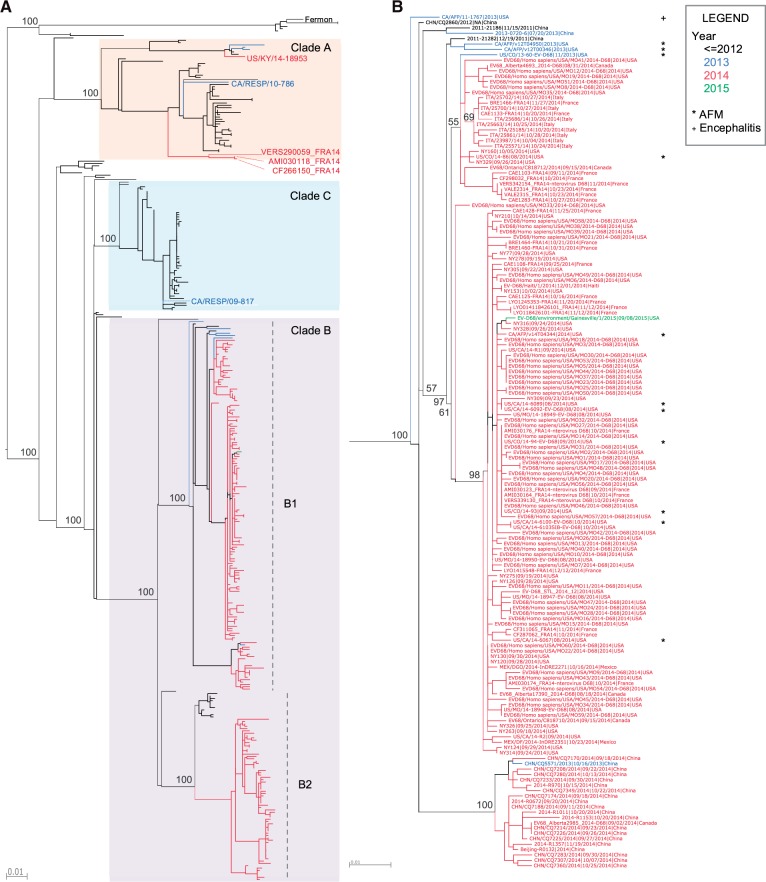
Lineage relationships of EV-D68 isolates inferred from VP1 phylogeny. (A) A phylogenetic tree of all available full-length VP1 nucleotide sequences as of 9 February 2016 in ViPR. The tree was constructed using RAxML and is color-coded by year of isolation (e.g. 2013: blue; 2014: red; 2015: green). Syntax for tree leaf labels is: strain name|isolation date|isolation country. Bootstrap support values are shown for all major branching nodes. Clade classifications are based on bootstrap values of 100 percent. Three major clades (A, B, and C) are present ( Tokarz et al. 2012), with clade B split into subclades B1 and B2. (B) A close-up of subclade B1. Isolates associated with AFM/AFP and encephalitis (Greninger et al. 2015) are marked with an asterisk and a plus sign, respectively.

To explore the evolutionary origin of the recent EV-D68 isolates since its initial detection in 1962 (Schieble et al. 1967), a phylogenetic tree was constructed using EV-D68 VP1 full-length nucleotide sequences. VP1 was chosen because it is the most variable protein in EV-D68 and would, therefore, be the most informative for phylogenetic analysis, and has been the target of enterovirus genotyping and the focus of EV-D68 sequencing efforts historically. Recent EV-D68 isolates, from 2013 and 2014, are positioned within all three previously defined major clades – A, B, and C (Tokarz et al. 2012) (Fig. 1A). Both clades A and B contain recent isolates from North America, Europe, and Asia, suggesting that distinct EV-D68 clades were co-circulating in these continents during this time period. The majority of the recent US isolates, including those associated with acute flaccid myelitis/paralysis (AFM/AFP) cases (Greninger et al. 2015) and a series of recent isolates from Canada, Mexico, France, Italy, and China assemble into a tight cluster within clade B, which we have designated as subclade B1 (Fig. 1B).

### 3.2 Neurovirulent phenotype is correlated with the B1 phylogenetic cluster

Since all EV-D68 isolates associated with neurological illnesses belong to the B1 subclade, we tested the association between neurovirulence and phylogeny using the BaTS program (Parker et al. 2008). Each EV-D68 isolate is assigned either a neurovirulent or non-neurovirulent trait. The analysis results show that parsimony score (PS) and association index (AI) statistics, which both measure the degree to which isolates of the same trait cluster together in a phylogenetic tree, were significant (Table 1). The monophyletic clade (MC) statistic, which measures the size of the maximum monophyletic clade whose tips all share the same trait, was significant for both neurovirulent and non-neurovirulent traits. These data suggest that the neurovirulent phenotype is statistically correlated with phylogenetic structure. Since all neurovirulent isolates belong to the monophyletic group B1 defined by a bootstrap support value of 100 percent, downstream comparative analyses were conducted with an emphasis on the B1 subclade.

**Table 1.** Neurovirulence–phylogeny association analysis results

Statistic	Observed mean	Lower 95% CI	Upper 95% CI	Null mean	Lower 95% CI	Upper 95% CI	Significance
AI	1.6579	1.1240	2.2064	2.5287	2.0605	2.9795	0.0020
PS	10.1875	10.0000	11.0000	11.8886	11.3057	12.0000	0.0010
MC (neurovirulent)	2.0088	2.0000	2.0000	1.1065	1.0000	1.6895	0.0060
MC (non-neurovirulent)	82.7451	81.0000	83.0000	49.8731	27.6094	80.7979	0.0330

### 3.3 EV-D68 B1 isolates have acquired unique substitutions that are also observed in EV-D70, PV, and EV-A71 viruses

**Figure 2.**
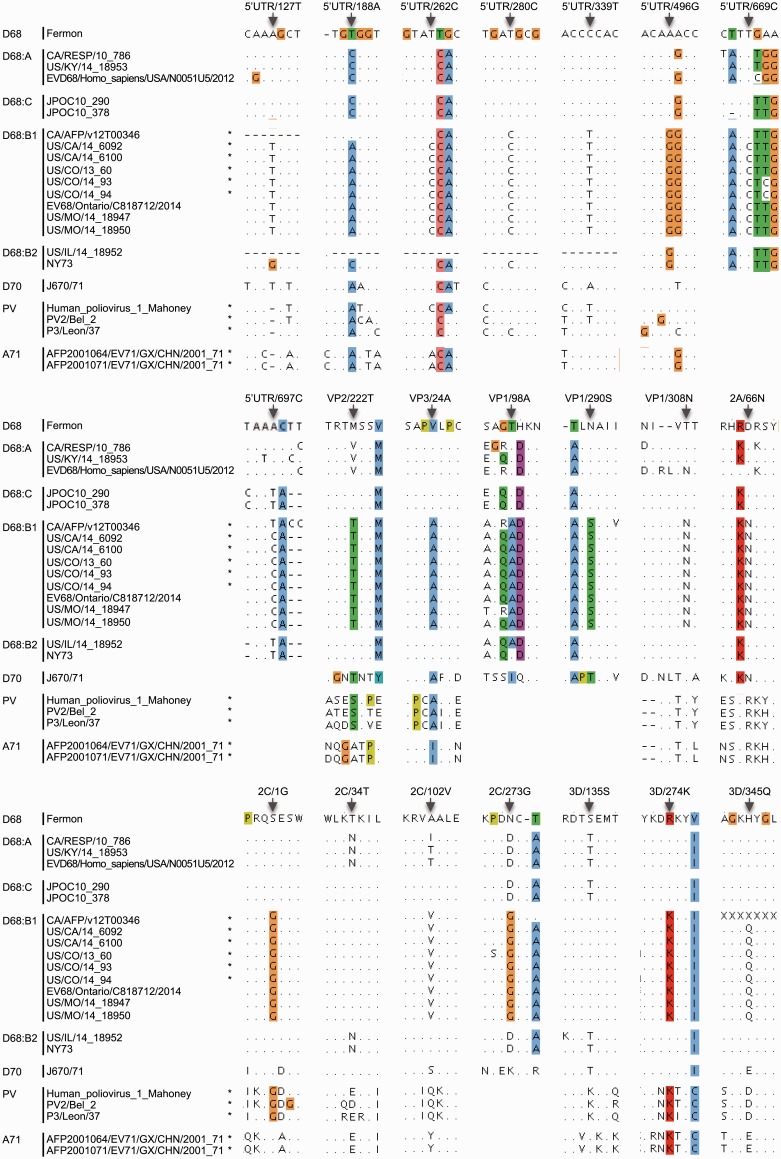
Unique substitutions in EV-D68 B1 subclade in comparison with representative isolates of non-B1 EV-D68, EV-D70, PV, and EV-A71 viruses. Isolates associated with AFM/AFP (Greninger et al. 2015) are marked with an asterisk. EV-D68 numbering is based on US/CO/13-60. Unique substitution positions are indicated by arrows. Alignments are highlighted using the Clustal × Color Scheme (Jalview); as a result, the colored residues are not necessarily at the reported substitution positions. Hypervariable regions are masked out.

To determine if there are any consistent substitutions that characterize subclade B1, which contains the AFM/AFP-associated isolates, we first used the ViPR meta-CATS statistical comparative analysis tool (Pickett et al. 2013) to analyze each mature peptide and the 5’UTR region. This analysis identified 117 amino acid positions in all mature peptides except 3B and 88 nucleotide positions in the 5′UTR that significantly differ between B1 and non-B1 isolates (Supplementary Table S1). Since the Chi-squared statistic used in the meta-CATS analysis does not take into account isolate dependency due to shared evolutionary ancestry, we conducted a second statistical test that specifically corrects for evolutionary correlation among isolates (Supplementary method). The second test identified a subset of meta-CATS positions that remained significant after controlling for isolate relatedness (Supplementary Table S1). Statistically significant positions were further filtered for high substitution sensitivity and specificity as defined in the Materials and Methods section, resulting in 21 positions that are diagnostic for subclade B1 of the 2014 outbreak (Table 2). These B1 distinct substitutions include 5′UTR/127T (the presence of a thymine at nucleotide position 127 in the 5′UTR), 5′UTR/188A, 5′UTR/262C, 5′UTR/280C, 5′UTR/339T, 5′UTR/496G, 5′UTR/669C, and 5′UTR/697C, and VP2/222T (the presence of a threonine at amino acid position 222 in the VP2 protein), VP3/24A, VP1/98A, VP1/290S, VP1/308N, 2A/66N, 2C/1G, 2C/34T, 2C/102V, 2C/273G, 3D/135S, 3D/274K, and 3D/345Q (Table 2 and Fig. 2).

**Table 2.** Unique substitutions in isolates from the EV-D68 B1 subclade in comparison with non-B1 EVD68 and their relationship with residues in PV, EV-D70, and EV-A71 viruses

EV-D68 genome region	Position (UTR: NT; mature peptides: AA)^a^	B1 EV-D68 NT/AA distribution	Non-B1 EV-D68 NT/AA distribution	*P* value^b^	B1 predominant NT/AA	Sensitivity (%)	Specificity (%)	PV-1/2/3 position and residue^c^		EV-D70 position and residue		EV-A71 position and residue		Observed in Other Viruses
5′UTR	127	10 G, 89 T	17 A, 5 G	6.25E−03	T	90	100	^	–	128	T	^	–	D70
5′UTR	188	90 A, 23 T	47 C, 17 T	2.13E−02	A	80	100	185	A	190	A	189	A	PV, D70, A71
5′UTR	262	91 C, 24 T	65 T	2.13E−02	C	79	100	259	C/T	264	T	264	A	PV
5′UTR	280	114 C, 1 T	8 C, 58 T	6.97E−03	C	99	88	277	C/T	282	T	#	–	PV
5′UTR	339	21 C, 94 T	65 C, 1 T	1.95E−02	T	82	98	337	C/T	341	A	338	C	PV
5′UTR	496	3 A, 112 G, 1 T	58 A, 10 G	1.12E−02	G	97	85	494	A	498	A	496	A	
5′UTR	669	92 C, 24 T	1 C, 54 T	2.19E−02	C	79	98	#	–	#	–	#	–	
5′UTR	697	1 A, 93 C, 22 T	36 A, 1 C, 18 T	2.03E−02	C	80	98	#	–	#	–	#	–	
VP2	222	22 M, 96 T	17 M, 18 V	1.22E−02	T	81	100	243	S	224	T	228	A	D70
VP3	24	96 A, 21 V	35 V	1.10E−02	A	82	100	24	A	24	A	24	I	PV, D70
VP1	98	155 A, 4 T	31 A, 181 T	1.60E−03	A	97	85	#	–	98	I	#	–	
VP1	290	27 N, 132 S	1 D, 211 N	9.58E−04	S	83	100	#	–	292	T	#	–	
VP1	308	134 N, 25 T	8 N, 204 T	1.61E−03	N	84	96	300	T	312	T	296	T	
2A	66	21 D, 101 N	32 D	7.50E−03	N	83	100	68	R	66	N	69	R	D70
2C	1	97 G, 20 S	32 S	9.36E−03	G	83	100	1	G	1	S	1	S	PV
2C	34	1 I, 1 N, 115 T	26 N, 6 T	2.35E−03	T	98	81	34	D/E	34	T	34	E	D70
2C	102	117 V	17 A, 1 I, 14 T	2.75E−04	V	100	100	102	Q	102	S	102	Y	
2C	273	3 D, 107 G, 7 S	26 D, 5 G, 1 N	8.74E−03	G	91	84	#	–	273	K	#	–	
3D	135	116 S	2 S, 30 T	4.78E−04	S	100	94	139	K	135	T	139	S	A71
3D	274	94 K, 22 R	32 R	1.16E−02	K	81	100	278	K	274	R	279	K	PV, A71
3D	345	18 H, 97 Q, 1 Y	32 H	9.14E−03	Q	84	100	349	D	345	E	350	E	

# , Hypervariable region.

^ , Gap. –, Not available.

^a^EV-D68 numbering is based on US/CO/13-60.

^b^*P* value from statistical analysis accounting for evolutionary correlation among isolates.

^c^PV numbering is based on PV-1 Mahoney NC_002058.

It is well established that other enteroviruses, especially PV, EV-D70, and EV-A71, are associated with paralysis symptoms (Pallansch and Roos 2006). To explore whether subclade B1 has acquired substitutions that are characteristic of these other viruses, we mapped the significantly different positions to the equivalent positions in representative sequences of these other enteroviruses. This comparison identified 12 of the 21 diagnostic positions in B1 where the same nucleotide or amino acid residues are observed at the equivalent positions in these other paralysis-causing enteroviruses (Table 2 andFig. 2), including 5′UTR/127T, VP2/222T, 2A/66N, and 2C/34T observed in EV-D70 only; 5′UTR/262C, 5′UTR/280C, 5′UTR/339T, and 2C/1G observed in PV only; 3D/135S observed in EV-A71 only; VP3/24A observed in EV-D70 and PV; 3D/274K observed in PV and EV-A71; 5′UTR/188A observed in EV-D70, PV and EV-A71.

## 4. Discussion

**Figure 3.**
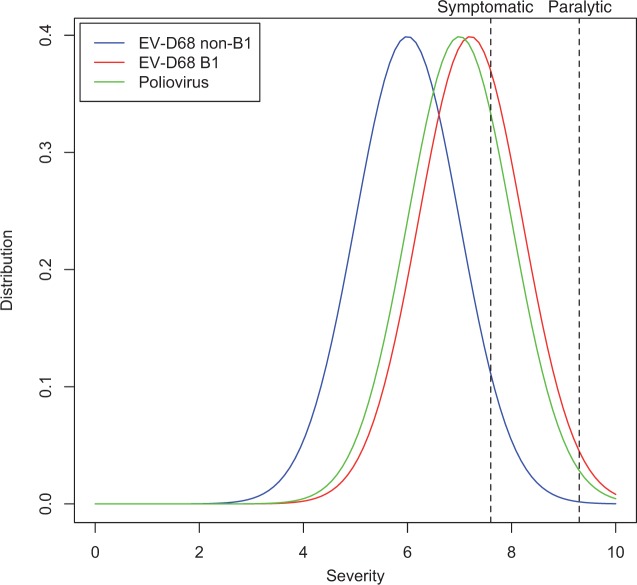
Conceptual model of enterovirus symptomatic and paralytic symptom distribution. Distributions of disease severity caused by B1, non-B1, and PV isolates are represented by hypothetical curves in red, blue, and green, respectively. For a given isolate lineage, disease severity would be influenced by the genetic background and co-morbidities of the infected individual. Symptomatic (28 percent) and paralytic (1 percent) threshold estimates represented by the black dashed lines are based on the clinical features of PV infections (CDC 2015a). See text for further discussion.

The manifestation of clinical symptoms following virus infection results from the combined effects of both virus and host factors. Most enterovirus infections are asymptomatic. However, when enterovirus infections are symptomatic, they can cause a spectrum of clinically distinct syndromes (Pallansch and Roos 2006). As an example, up to 72 percent of poliovirus infections are asymptomatic. The most common symptomatic disease caused by poliovirus is a mild febrile illness called abortive poliomyelitis, which occurs in approximately 24 percent of infected children. About 1–5 percent of poliovirus infected individuals develop non-paralytic aseptic meningitis, while fewer than 1 percent develop flaccid paralysis (CDC 2015b). We hypothesize that clinical manifestations of enterovirus infections in a susceptible population follow some type of severity distribution (Fig. 3). An infected person only presents symptoms when virus infection exceeds some symptomatic threshold, and presents paralytic syndromes when virus infection exceeds some paralytic threshold. Prior to the 2014 outbreak, EV-D68 was one of the most rarely reported enterovirus infections, with only 26 documented cases by the National Enterovirus Surveillance System in the United States from 1970 to 2005 (Khetsuriani et al. 2006). The 2014 EV-D68 outbreak is unprecedented in its much larger scale, increased disease severity, and more prevalent neurological symptoms. One possible explanation is that disease severity and neuropathology during a D68 infection could be quantitative traits and that the genetic changes acquired during the establishment of the B1 subclade have caused a shift in these traits toward increased severity (Fig. 3, red curve). However, even with this shift toward increased severity, this quantitative trait may or may not exceed some clinical thresholds (Fig. 3, dashed lines) depending on the genetic background and co-morbidities of the infected individual, which would explain why some subclade B1 isolates were obtained from patients without evidence of neurovirulence. From the perspective of paralytic symptoms, the incidence of paralysis in symptomatic poliovirus infections is ∼3.6 percent (1 percent paralysis/28 percent symptomatic infections), while the incidence of paralysis in symptomatic EV-D68 subclade B1 infection is estimated to be 6.9 percent (11 AFP cases and assuming that all the 159 B1 isolates studied here represent a reasonable estimate of the total symptomatic case load during the same time period), almost twice the rate of poliovirus infections. However, the available data on clinical presentations of EV-D68 infections are incomplete and biased, and so any current estimates of symptomatic incidence are crude at best.

The phylogenetic and phenotypic association analysis results obtained using BaTS clearly indicates that the neuropathology phenotype is correlated with phylogenetic clustering. Since all neurological illness-associated isolates belong to the monophyletic subclade B1, we hypothesize that this subclade has acquired genetic changes enhancing neurotropism of EV-D68. Comparative genomic analyses reveal that 12 B1 diagnostic positions carry the same residues as those observed at equivalent positions in other enteroviruses including poliovirus, EV-D70, and/or EV-A71. Since these enteroviruses are known to cause paralysis symptoms more frequently, one or more of these substitutions found in the B1 subclade could be linked to a more neurovirulent phenotype, perhaps by enabling EV-D68 to replicate more efficiently in neuronal cells. This hypothesis is currently being tested in a variety of different cell culture model systems.

The mechanisms of pathogenicity and cellular tropism of EV-D68 are not fully understood. Capsid proteins, non-structural proteins, and UTR regions may all affect EV-D68 infectivity, replication efficiency, and pathogenicity in different host tissues and cell types (Gromeier et al. 1996; Lin and Shih 2014). Our study has pinpointed a series of B1-unique substitutions residing in the VP1, VP2, and VP3 capsid proteins, the 2A, 2C, and 3D non-structural proteins, and the internal ribosome entry sequence (IRES). Among these, VP2/222T, VP3/24A, VP1/308N, 2A/66N, 2C/1G, and 3D/274K were also identified in a previous report (Greninger et al. 2015).

Picornavirus entry into cells begins with the attachment of viral proteins to host receptors. Mapping the locations of B1 substitutions on a capsid protein structure shows that VP1/290S, VP1/308N, and VP2/222T are located on the surface of the virion (Supplementary Fig. S1A) and could be directly involved in virus–host cell attachment. Most infectious enterovirus virions contain a small hydrophobic molecule that appears to stabilize the virus by filling the VP1 pocket. When a receptor binds to the virus, conformational change in the binding region releases the hydrophobic molecule and consequently initiates uncoating of the virion (Liu et al. 2015). Antiviral drugs developed for related enteroviruses, including pleconaril, replace this endogenous hydrophobic molecule and thereby inhibit uncoating of the virion (Lin and Shih 2014). VP3/24A is one of the sites that comprise this VP1 pocket (Supplementary Fig. S1B). Substitutions in this site could potentially affect the stability of the virion and/or the uncoating process.

Enterovirus genomes encode a single open reading frame that is translated into one long polyprotein, which is subsequently cleaved by viral proteases (3C and 2A) into functional mature peptides. Substitution 2C/1G is distinct in subclade B1 and also observed in poliovirus (Table 2 andFig. 2). This position is located in the 2B↓2C (P1↓P1’) cleavage site for the 3C cysteine protease. 3C of many picornaviruses has less strict cleavage specificities, with the structures and sequences surrounding the cleavage site influencing cleavage rates (Racaniello 2006). A study on the substrate specificity of EV-D68 3C found that in general, Gly in the P1’ site leads to higher cleavage activities than Ser or Leu (Tan et al. 2013). Thus, the S-to-G substitution in P1’ position of the 2B↓2C cleavage site of subclade B1 could improve cleavage efficiency between 2B and 2C. Interestingly, substitution VP1/308N is immediately adjacent to the P1 position in the VP1↓2A cleavage site. Whether this substitution also affects cleavage efficiency is unclear.

Picornaviruses have a conserved and highly structured 5′UTR region, indicating the importance of this region in the virus life cycle (Rivera et al. 1988; Stewart and Semler 1997). An internal ribosome entry site (IRES) is located in the 5’UTR of picornaviruses and facilitates cap-independent translation. Interestingly, virulence determinants have been found in the IRES element of poliovirus (Evans et al. 1985; Guillot et al. 1994; Rezapkin et al. 1999) and EV-A71 virus (Yeh et al. 2011). In the attenuated polio vaccine strain Sabin, sequence alterations in the IRES were found to specifically attenuate IRES function in neuronal cells but not other cell types (Campbell et al. 2005), suggesting that cell type-specific IRES function may be partially responsible for neurovirulence of wild type poliovirus. Our study identified six substitutions located in the IRES element of all or the majority of subclade B1 isolates (Supplementary Fig. S2). The functional relevance of these substitutions is being tested in a variety of different cell culture model systems.

In some cases, not all AFM/AFP isolates carry the same substitutions (e.g. 5′UTR/669T in the CA/AFP/v12T00346 isolate). The analysis performed using BaTS allows us to reject the null hypothesis that there is no association between the neurovirulence trait and the phylogenetic topology, so we based the polymorphism analysis on subclade B1, which includes all AFM/AFP isolates. However, there are exceptions to a strict residue association. For example, the CA/AFP/v12T00346 isolate differs from the other AFM/AFP isolates at four positions in the 5′UTR region (Fig. 2). This strain resembles clade A at the 3′ end of the 5’UTR and may be a recombinant strain. The other four 5′UTR substitutions and all amino acid substitutions that differ between B1 and other D68 isolates are conserved among all AFM/AFP isolates, including 5′UTR/127T, 5′UTR/280C, 5′UTR/339T, 5′UTR/496G, VP2/222T, VP3/24A, VP1/98A, VP1/290S, VP1/308N, 2A/66N, 2C/1G, 2C/34T, 2C/102V, 2C/273G, 3D/135S, 3D/274K, and 3D/345Q.

In summary, our results suggest that three EV-D68 clades were co-circulating in 2013–2014; isolates associated with AFM/AFP belong to a distinct phylogenetic cluster designated as B1; the majority of the B1 isolates have 21 unique nucleotide and amino acid substitutions that distinguish them from other EV-D68 isolates, among which 12 are observed at equivalent positions in other enteroviruses associated with neurovirulent phenotypes. Based on these results, we hypothesis that unique B1 substitutions might contribute to the increased incidence of neuropathology associated with the 2014 outbreak.

## 4.1 Data availability

All sequences are available in the Virus Pathogen Resource (ViPR) – *Picornaviridae* family (http://www.viprbrc.org/brc/home.spg?decorator=picorna).

## Supplementary data

Supplementary data are available at Virus Evolution online.

## Supplementary Material

Supplementary Data
