# Supplementary material for: Genetic changes found in a distinct clade of Enterovirus D68 associated with paralysis during the 2014 outbreak
Source: Virus Evol. 2016 Jun 15;2(1):vew015. doi: 10.1093/ve/vew015 (PMC5426007; doi:10.1093/ve/vew015)
Supplement: Supplementary Data [file vew015_Supp.zip › vew015-suppl_data/D68_comparative_analysis_manuscript_supp_figures_13MAR2016_submission.pdf]

Figure S1. Locations of B1-unique substitutions in capsid protein structure (PDB ID: 4WM7)

(A) Space filling view of EV-D68 capsid proteins. (B) Secondary structure view of EV-D68 capsid proteins. Positions of B1-unique substitutions are colored in purple. VP1/290 (corresponding to VP1/278 in the structure) is located on the surface and close to putative receptor binding site [25]. VP3/24 is located within the VP1 pocket (colored in blue) [25] near the binding site for the anti-viral drug pleconaril (colored in cyan).

Figure S2. Locations of B1-unique substitutions in 5'UTR structure

5'UTR structure of poliovirus reported by Stewart and Semler [28]. Mapping of EV-D68 B1-unique substitutions to this structure shows that six substitutions (127U, 188A, 262C, 280C, 339T, and 496G) are positioned in the IRES element: 127U and 188A (corresponding to 123 and 185 on PV-1 Mahoney, respectively) are positioned one nucleotide upstream of the stem loop structures II and III with the substitutions predicted to add an additional base pair at the base of these stems; 262C, 280C, and 339T are positioned within stem loop structure IV; 496G is positioned within stem loop V. EV-D68 numbering is based on US/CO/13-60.

Figure S1

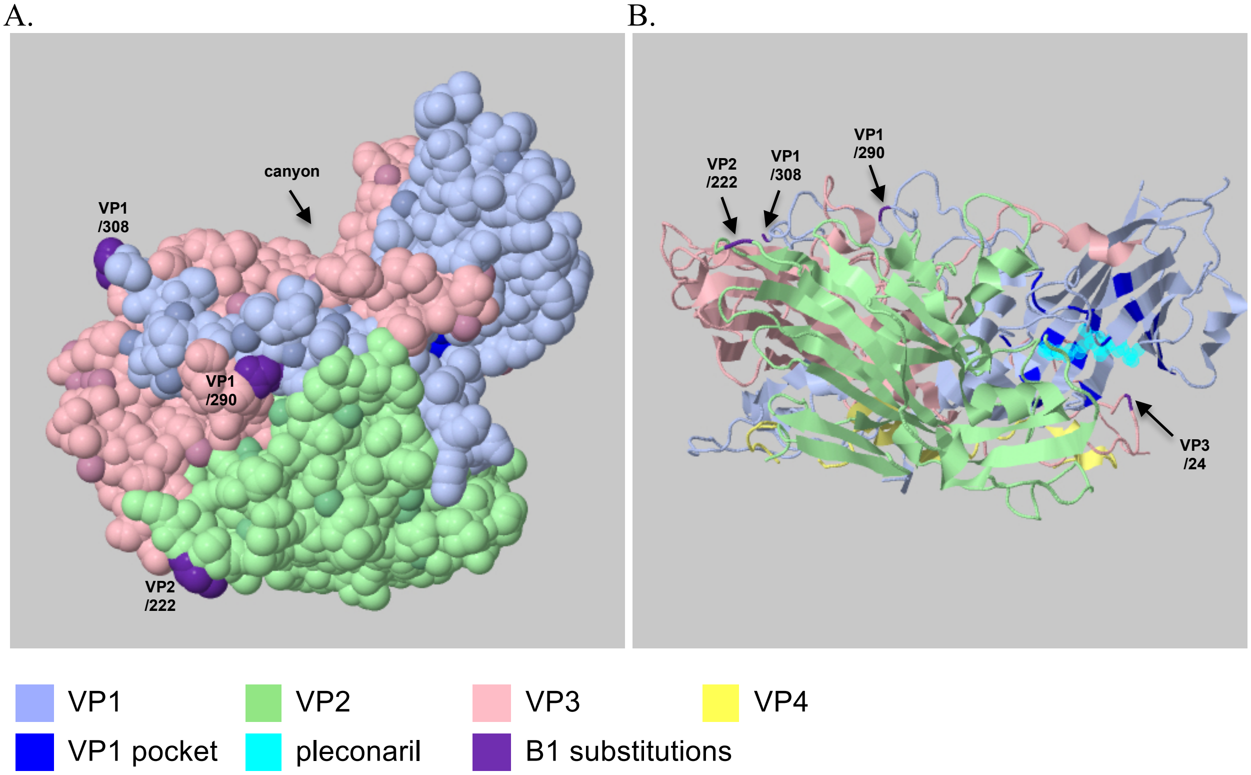

A.
B.
canyon
VP1 /308
VP1 /290
VP2 /222
VP2 /222
VP1 /308
VP1 /290
VP3 /24
VP1
VP2
VP3
VP4
VP1 pocket
pleconaril
B1 substitutions

Figure S2

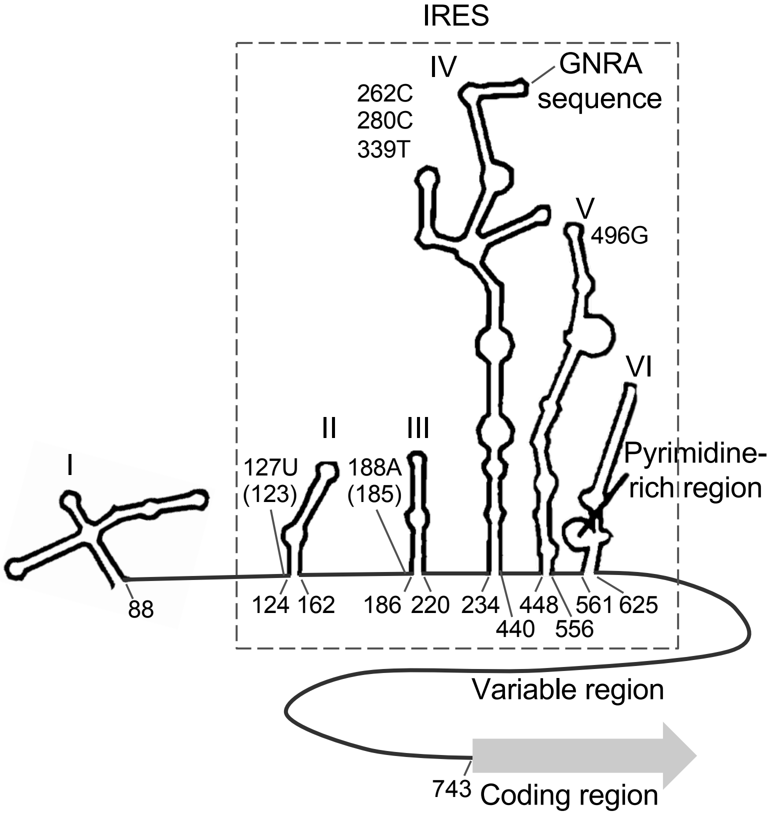
IRES
IV
GNRA
sequence
262C
280C
339T
V
496G
VI
II
III
I
127U
(123)
188A
(185)
Pyrimidine-
rich region
88
124
162
186
220
234
440
448
556
561
625
Variable region
743
Coding region

Table S1. Unique substitutions in EV-D68 B1 subclade in comparison with non-B1 isolates

EV-D68 Genome Region	Position (UTR: NT; Mature Peptides: AA)[a]	B1 EV-D68 NT/AA Distribution	Non-B1 EV-D68 NT/AA Distribution	B1 Predominant NT/AA	P-value[b]	Sensitivity	Specificity
5UTR	28	3 C, 87 T	1 -, 14 C, 5 T	T	1.20E-02	97%	75%
5UTR	62	95 C	7 C, 15 T	C	6.15E-03	100%	68%
5UTR	63	96 T	2 C, 20 T	T		100%	9%
5UTR	89	2 C, 96 T	2 A, 20 T	T		98%	9%
5UTR	90	98 C	20 C, 2 T	C		100%	9%
5UTR	96	98 T	8 C, 14 T	T		100%	36%
5UTR	102	98 T	2 C, 20 T	T		100%	9%
5UTR	115	10 C, 88 T	9 C, 13 T	T		90%	41%
5UTR	117	97 C, 1 T	9 C, 13 T	C	1.52E-02	99%	59%
5UTR	119	98 T	16 C, 6 T	T	4.09E-03	100%	73%
5UTR	125	99 A	16 A, 6 G	A		100%	27%
5UTR	127	10 G, 89 T	17 A, 5 G	T	6.25E-03	90%	100%
5UTR	133	98 A, 1 G	17 A, 5 G	A		99%	23%
5UTR	141	1 A, 99 G	16 A, 6 G	G	5.04E-03	99%	73%
5UTR	148	112 A	18 A, 5 G	A		100%	22%
5UTR	157	111 C, 1 T	11 C, 14 T	C	1.15E-02	99%	56%
5UTR	158	112 T	5 C, 20 T	T		100%	20%
5UTR	159	111 C, 1 T	19 C, 6 T	C		99%	24%
5UTR	160	112 A	20 A, 5 G	A		100%	20%
5UTR	169	112 C	22 C, 3 T	C		100%	12%
5UTR	173	111 C, 1 T	11 C, 14 T	C	1.15E-02	99%	56%
5UTR	178	110 C, 2 T	9 C, 16 T	C	8.35E-03	98%	64%
5UTR	188	90 A, 23 T	47 C, 17 T	A	2.13E-02	80%	100%
5UTR	198	113 A	26 A, 38 G	A	3.67E-02	100%	59%
5UTR	204	113 T	9 C, 55 T	T		100%	14%
5UTR	205	113 C	38 C, 26 T	C		100%	41%
5UTR	223	114 C	31 C, 34 T	C		100%	52%
5UTR	233	1 A, 1 C, 113 T	7 A, 1 C, 57 T	T		98%	12%
5UTR	262	91 C, 24 T	65 T	C	2.13E-02	79%	100%
5UTR	273	115 T	5 G, 61 T	T		100%	8%
5UTR	280	114 C, 1 T	8 C, 58 T	C	6.97E-03	99%	88%
5UTR	319	115 T	19 C, 47 T	T		100%	29%
5UTR	320	115 C	45 C, 21 T	C		100%	32%
5UTR	334	97 A, 18 G	30 A, 1 C, 35 G	A		84%	55%
5UTR	339	21 C, 94 T	65 C, 1 T	T	1.95E-02	82%	98%
5UTR	346	111 C, 4 T	29 C, 37 T	C		97%	56%
5UTR	389	35 A, 81 G	64 A, 2 G	G		70%	97%
5UTR	403	116 G	9 A, 57 G	G		100%	14%
5UTR	472	116 C	16 C, 52 T	C	1.28E-02	100%	76%
5UTR	496	3 A, 112 G, 1 T	58 A, 10 G	G	1.12E-02	97%	85%
5UTR	497	116 G	16 A, 52 G	G		100%	24%
5UTR	504	116 A	61 A, 9 G	A		100%	13%
5UTR	519	1 C, 115 T	26 C, 44 T	T		99%	37%
5UTR	574	116 T	33 C, 22 T	T	2.52E-02	100%	60%
5UTR	601	1 A, 113 G, 2 T	34 A, 21 G	G	3.41E-02	97%	62%
5UTR	621	116 T	6 A, 49 T	T		100%	11%

5UTR	626	116 A	31 A, 24 G	A		100%	44%
5UTR	629	104 C, 12 T	2 A, 24 C, 29 T	C		90%	56%
5UTR	633	2 C, 114 T	39 C, 16 T	T	1.70E-02	98%	71%
5UTR	634	8 A, 108 G	28 A, 27 G	G		93%	51%
5UTR	636	115 A, 1 G	26 A, 29 G	A	4.46E-02	99%	53%
5UTR	637	116 A	19 A, 2 C, 34 T	A	1.81E-02	100%	65%
5UTR	638	115 A, 1 G	28 A, 27 G	A		99%	49%
5UTR	640	115 G, 1 T	34 A, 21 G	G	2.61E-02	99%	62%
5UTR	641	116 A	49 A, 6 G	A		100%	11%
5UTR	642	1 A, 115 T	5 -, 9 C, 41 T	T		99%	25%
5UTR	644	114 C, 2 T	46 C, 9 T	C		98%	16%
5UTR	645	116 T	2 A, 2 C, 1 G, 50 T	T		100%	9%
5UTR	648	82 A, 32 C, 2 G	51 A, 4 G	A		71%	7%
5UTR	651	116 T	5 C, 50 T	T		100%	9%
5UTR	653	116 A	39 A, 16 G	A		100%	29%
5UTR	655	116 T	7 C, 48 T	T		100%	13%
5UTR	656	4 C, 112 T	12 C, 43 T	T		97%	22%
5UTR	658	18 C, 98 T	6 A, 11 C, 38 T	T		84%	31%
5UTR	662	105 C, 11 T	19 C, 1 G, 35 T	C		91%	65%
5UTR	664	116 T	5 C, 50 T	T		100%	9%
5UTR	665	116 G	7 A, 48 G	G		100%	13%
5UTR	666	114 C, 2 T	1 A, 25 C, 29 T	C	4.61E-02	98%	55%
5UTR	667	116 A	1 -, 47 A, 1 G, 6 T	A		100%	15%
5UTR	669	92 C, 24 T	1 C, 54 T	C	2.19E-02	79%	98%
5UTR	670	116 T	13 C, 2 G, 40 T	T		100%	27%
5UTR	671	14 C, 102 T	2 A, 34 G, 19 T	T		88%	65%
5UTR	676	116 A	23 A, 32 G	A	2.80E-02	100%	58%
5UTR	680	2 C, 114 T	44 C, 11 T	T	9.36E-03	98%	80%
5UTR	681	93 A, 23 G	54 A, 1 G	A		80%	2%
5UTR	682	1 -, 113 A, 2 G	27 -, 17 A, 11 T	A		97%	69%
5UTR	684	116 T	5 C, 50 T	T		100%	9%
5UTR	685	4 C, 112 T	5 A, 50 T	T		97%	9%
5UTR	687	114 A, 2 T	50 A, 5 G	A		98%	9%
5UTR	688	116 A	48 A, 7 G	A		100%	13%
5UTR	689	116 A	38 A, 17 G	A		100%	31%
5UTR	691	116 T	5 C, 2 G, 48 T	T		100%	13%
5UTR	693	1 C, 115 T	31 C, 24 T	T	3.61E-02	99%	56%
5UTR	694	6 C, 110 T	6 -, 4 C, 45 T	T		95%	18%
5UTR	697	1 A, 93 C, 22 T	36 A, 1 C, 18 T	C	2.03E-02	80%	98%
5UTR	698	115 A, 1 C	19 A, 34 C, 2 T	A	2.09E-02	99%	65%
5UTR	^	1 A, 1 T	36 A	NA	2.40E-02	N/A	N/A
5UTR	^	1 A, 1 T	34 A, 1 C, 1 T	NA	2.40E-02	N/A	N/A
VP4	18	100 I, 18 V	35 I	I		85%	0%
VP4	65	118 A	31 A, 4 V	A		100%	11%
VP2	73	22 A, 96 T	14 A, 1 I, 1 S, 19 T	T		81%	46%
VP2	74	118 G	18 E, 15 G, 2 N	G	1.16E-02	100%	57%
VP2	98	118 H	24 H, 11 Y	H		100%	31%
VP2	135	116 H, 2 Y	18 H, 17 Y	H	3.42E-02	98%	49%
VP2	136	118 N	3 D, 32 N	N		100%	9%
VP2	142	1 E, 117 G	4 E, 27 G, 2 R, 2 W	G		99%	23%

VP2	144	117 D, 1 T	2 A, 20 D, 3 N, 10 T	D	4.08E-02	99%	43%
VP2	151	18 A, 100 E	33 E, 2 R	E		85%	6%
VP2	156	118 N	24 N, 11 S	N		100%	31%
VP2	211	118 I	31 I, 4 V	I		100%	11%
VP2	222	22 M, 96 T	17 M, 18 V	T	1.22E-02	81%	100%
VP2	247	2 A, 116 T	5 A, 30 T	T		98%	14%
VP3	24	96 A, 21 V	35 V	A	1.10E-02	82%	100%
VP3	46	117 V	2 I, 2 M, 31 V	V		100%	11%
VP3	47	3 I, 114 V	8 I, 27 V	V		97%	23%
VP3	60	1 G, 115 S, 1 T	1 D, 2 G, 16 N, 16 S	S	2.44E-02	98%	54%
VP3	65	117 E	17 E, 18 Q	E	1.87E-02	100%	51%
VP3	73	117 A	15 A, 20 V	A	1.25E-02	100%	57%
VP3	153	115 I, 2 V	26 I, 9 V	I		98%	26%
VP3	166	117 I	19 I, 16 V	I	2.60E-02	100%	46%
VP3	180	117 N	1 G, 33 N, 1 S	N		100%	6%
VP3	204	118 S	3 N, 32 S	S		100%	9%
VP3	208	119 D	17 D, 18 N	D	1.54E-02	100%	51%
VP3	216	121 I	17 I, 18 V	I	1.35E-02	100%	51%
VP3	234	120 G, 1 R	1 A, 32 G, 2 R	G		99%	9%
VP1	1	159 L	75 I, 135 L, 2 S	L		100%	36%
VP1	2	157 D, 2 N	201 D, 8 E, 3 N	D		99%	5%
VP1	5	158 H, 1 Q	115 D, 97 H	H	2.79E-02	99%	54%
VP1	6	145 A, 14 G	200 A, 6 E, 6 G	A		91%	6%
VP1	46	159 T	59 S, 153 T	T		100%	28%
VP1	76	159 S	8 G, 204 S	S		100%	4%
VP1	90	159 D	84 D, 128 N	D	1.57E-02	100%	60%
VP1	92	159 T	50 A, 161 T, 1 V	T		100%	24%
VP1	95	158 A, 1 T	81 A, 2 D, 119 E, 1 G, 1 K, 6 S, 2 V	A	1.51E-02	99%	62%
VP1	97	155 Q, 4 R	6 G, 168 Q, 38 R	Q		97%	21%
VP1	98	155 A, 4 T	31 A, 181 T	A	1.60E-03	97%	85%
VP1	99	159 D	204 D, 8 H	D		100%	4%
VP1	110	2 K, 157 R	67 K, 145 R	R		99%	32%
VP1	131	159 I	166 I, 46 V	I		100%	22%
VP1	140	159 N	52 -, 159 N, 1 S	N		100%	25%
VP1	141	1 D, 158 G	8 D, 162 G, 42 S	G		99%	24%
VP1	143	10 G, 1 N, 148 S	14 N, 196 S, 2 T	S		93%	8%
VP1	144	6 -, 152 N, 1 S	7 D, 149 N, 56 S	N		96%	30%
VP1	145	158 N, 1 S	147 N, 64 S, 1 T	N		99%	31%
VP1	146	159 T	3 A, 2 R, 20 S, 187 T	T		100%	12%
VP1	148	1 A, 3 M, 155 V	3 A, 100 M, 39 T, 70 V	V	1.21E-02	97%	67%
VP1	152	159 D	201 D, 11 N	D		100%	5%
VP1	168	159 E	148 E, 64 K	E		100%	30%
VP1	169	159 K	6 E, 148 K, 56 Q, 2 R	K		100%	30%
VP1	178	159 G	43 A, 168 G, 1 R	G		100%	21%
VP1	187	159 I	201 I, 1 T, 10 V	I		100%	5%
VP1	194	101 I, 58 M	1 I, 211 M	I	7.17E-03	64%	100%
VP1	207	2 I, 157 V	53 I, 1 T, 158 V	V		99%	25%
VP1	218	102 N, 57 S	38 N, 174 S	N		64%	82%
VP1	243	159 V	123 I, 89 V	V	1.91E-02	100%	58%
VP1	269	159 L	204 L, 8 M	L		100%	4%

VP1	280	159 K	95 K, 117 R	K	2.39E-02	100%	55%
VP1	283	159 E	34 D, 171 E, 5 K, 2 Y	E		100%	19%
VP1	284	159 R	3 K, 203 R, 6 T	R		100%	4%
VP1	290	27 N, 132 S	1 D, 211 N	S	9.58E-04	83%	100%
VP1	297	159 D	6 A, 188 D, 13 E, 3 G, 2 N	D		100%	11%
VP1	305	159 N	9 D, 202 N, 1 S	N		100%	5%
VP1	^	159 -	204 -, 8 R	-		100%	4%
VP1	^	159 -	204 -, 8 L	-		100%	4%
VP1	308	134 N, 25 T	8 N, 204 T	N	1.61E-03	84%	96%
2A	22	85 A, 37 T	3 I, 29 T	A	3.16E-02	70%	100%
2A	25	1 K, 121 R	3 K, 29 R	R		99%	9%
2A	37	1 I, 1 L, 120 V	8 I, 24 V	V		98%	25%
2A	65	121 K, 1 R	23 K, 9 R	K		99%	28%
2A	66	21 D, 101 N	32 D	N	7.50E-03	83%	100%
2A	67	122 R	5 K, 27 R	R		100%	16%
2A	71	120 I, 1 V	19 I, 13 V	I	3.41E-02	99%	41%
2A	84	120 N, 1 S	23 N, 9 S	N		99%	28%
2A	99	121 V	20 A, 1 T, 11 V	V	3.42E-03	100%	66%
2A	144	111 A, 7 V	25 A, 7 V	A		94%	22%
2B	26	117 K	28 K, 4 R	K		100%	13%
2B	30	117 D	25 D, 7 N	D		100%	22%
2B	64	2 I, 115 V	5 I, 27 V	V		98%	16%
2C	1	97 G, 20 S	32 S	G	9.36E-03	83%	100%
2C	30	117 N	27 N, 5 S	N		100%	16%
2C	34	1 I, 1 N, 115 T	26 N, 6 T	T	2.35E-03	98%	81%
2C	58	104 N, 13 S	1 K, 20 N, 11 S	N		89%	38%
2C	61	2 N, 115 S	1 G, 5 N, 26 S	S		98%	19%
2C	102	117 V	17 A, 1 I, 14 T	V	2.75E-04	100%	100%
2C	154	117 I	17 I, 15 V	I	1.99E-02	100%	47%
2C	273	3 D, 107 G, 7 S	26 D, 5 G, 1 N	G	8.74E-03	91%	84%
2C	317	2 I, 115 T	8 I, 24 T	T		98%	25%
3A	8	117 I	26 I, 6 V	I		100%	19%
3A	16	1 I, 116 T	2 A, 1 I, 29 T	T		99%	9%
3A	46	39 I, 78 V	32 I	V	4.61E-02	67%	100%
3A	53	78 I, 39 L	30 L, 2 V	I	4.61E-02	67%	100%
3A	68	117 I	26 I, 6 V	I		100%	19%
3C	17	116 I	27 I, 5 V	I		100%	16%
3C	30	116 V	15 I, 17 V	V	2.14E-02	100%	47%
3C	31	116 Y	1 F, 8 H, 23 Y	Y		100%	28%
3C	77	116 L	25 L, 7 M	L		100%	22%
3C	92	114 R, 2 S	4 K, 28 R	R		98%	13%
3D	6	116 S	18 N, 14 S	S	9.92E-03	100%	56%
3D	17	116 A	28 A, 4 V	A		100%	13%
3D	41	115 P, 1 S	11 P, 21 S	P	6.38E-03	99%	66%
3D	44	116 P	1 L, 30 P, 1 S	P		100%	6%
3D	87	1 N, 115 S	2 R, 30 S	S		99%	6%
3D	88	116 V	14 I, 18 V	V	2.51E-02	100%	44%
3D	130	116 H	14 H, 18 Q	H	9.92E-03	100%	56%
3D	132	116 R	3 K, 29 R	R		100%	9%
3D	135	116 S	2 S, 30 T	S	4.78E-04	100%	94%

3D	139	116 K	16 K, 16 R	K	1.58E-02	100%	50%
3D	161	2 K, 114 R	4 K, 28 R	R		98%	13%
3D	196	116 N	16 N, 16 S	N	2.09E-02	100%	50%
3D	212	116 I	24 I, 1 M, 7 V	I		100%	25%
3D	222	115 D, 1 N	25 D, 7 N	D		99%	22%
3D	274	94 K, 22 R	32 R	K	1.16E-02	81%	100%
3D	345	18 H, 97 Q, 1 Y	32 H	Q	9.14E-03	84%	100%
3D	349	116 V	6 I, 6 L, 1 T, 19 V	V	3.35E-02	100%	41%
3D	360	1 A, 80 I, 35 V	1 I, 31 V	I		69%	97%

[a] EV-D68 numbering is based on US/CO/13-60.

[b] P-value from statistical analysis accounting for evolutionary correlation among isolates.

^ Gap

- Not available
