# Supplementary material for: Genetic changes found in a distinct clade of Enterovirus D68 associated with paralysis during the 2014 outbreak
Source: Virus Evol. 2016 Jun 15;2(1):vew015. doi: 10.1093/ve/vew015 (PMC5426007; doi:10.1093/ve/vew015)
Supplement: Supplementary Data [file vew015_Supp.zip › vew015-suppl_data/D68_supplement_statistical_test.pdf]

A Statistical Test on Comparing Two proportions with Correlated Observations

In this document, we describe the method used in the paper to detect mutations associated with EV-D68 B.1.2 membership while accounting for correlation among isolates due to common ancestry. Using the context of the paper, we use $w_i = 1/0$ $(i = 1, \cdots, n)$ to indicate whether the ith isolate is a member of the EV-D68 B.1.2 cluster, and $y_{ij} = 1/0$ to indicate whether there is a mutation at the jth $(j = 1, \cdots, J)$ position of isolate i. To assess the association between mutation and B.1.2 membership, the hypothesis of interest is $H_0 : p_j^{(0)} = p_j^{(1)}$ vs $H_1 : p_j^{(0)} \neq p_j^{(1)}$. Here $p_j^{(1)}$ is the probability of having a mutation at the jth position among isolates in the B.1.2 cluster. i.e., $p_j^{(1)} = E(y_{ij})$ for $w_i = 1$. The proportion $p_j^{(0)}$ is similarly defined for $w_i = 0$.

As a binary random variable, the first two moments of y_{ij} are $E(y_{ij}) = p_j^{(w_i)}$ and $\mathrm{Var}(y_{ij}) = p_j^{(w_i)}(1 - p_j^{(w_i)})$. Define $\boldsymbol{y}_j = (y_{1j}, \cdots, y_{nj})'$. Because the isolates are correlated due to the shared ancestry inherent in the phylogenetic tree, we use $\boldsymbol{R} = \mathrm{Corr}(\boldsymbol{y}_j, \boldsymbol{y}_j)$ to indicate the correlation among isolates which is assumed to be the same across all the positions. At each position, the sample mean for each group ($w = 0/1$ for non-cluster / cluster) is

$$\bar{y}_j^{(w)} = \sum_{i:w_i=w} y_{ij}/n_w \quad \text{for } w = 0, 1.$$

Here n_w is the number of isolates with $w_i = w$. Note that $\bar{y}_j^{(w)}$ is the sample estimate of $p_j^{(w)}$. According to the law of large numbers, $\bar{y}_j^{(1)} - \bar{y}_j^{(0)}$ approximately follows a normal distribution

$N(p_j^{(1)} - p_j^{(0)}, Var(\bar{y}_j^{(1)} - \bar{y}_j^{(0)}))$, where $Var(\bar{y}_j^{(1)} - \bar{y}_j^{(0)})$ denotes the true variance of $\bar{y}_j^{(1)} - \bar{y}_j^{(0)}$.

We test the null hypothesis $H_0 : p_j^{(0)} = p_j^{(1)}$ based on the statistic

$$Z_j = \frac{\bar{y}_j^{(1)} - \bar{y}_j^{(0)}}{\sqrt{\hat{Var}(\bar{y}_j^{(1)} - \bar{y}_j^{(0)})}},$$

with $\hat{Var}(\bar{y}_j^{(1)} - \bar{y}_j^{(0)})$ being the estimated variance under H_0. H_0 is rejected if $|Z_j| > z_{1-\alpha/2}$, where α is the two-sided significance level and $z_{1-\alpha/2}$ is the $100(1-\alpha/2)$th percentile of the standard normal distribution.

With independent observations, $\hat{Var}(\bar{y}_j^{(1)} - \bar{y}_j^{(0)}) = \bar{y}_j(1-\bar{y}_j)(1/n_0 + 1/n_1)$, where $\bar{y}_j = \sum_i y_{ij}/n$ is the estimated common probability under H_0. With dependent observations, $\hat{Var}(\bar{y}_j^{(1)} - \bar{y}_j^{(0)})$ is much more complicated. In the following, we outline its derivation. First, $\bar{y}_j^{(1)} - \bar{y}_j^{(0)}$ can be written as a linear combination of $\boldsymbol{y}_j$:

$$\bar{y}_j^{(1)} - \bar{y}_j^{(0)} = \boldsymbol{h}'\boldsymbol{y}_j,$$

where $\boldsymbol{h} = (h_1, \cdots, h_n)'$ with $h_i = 1/n_1$ if $w_i = 1$ and $h_i = -1/n_0$ if $w_i = 0$. This expression leads to $Var(\bar{y}_j^{(1)} - \bar{y}_j^{(0)}) = \boldsymbol{h}'\text{Cov}(\boldsymbol{y}_j, \boldsymbol{y}_j)\boldsymbol{h}$. Note that under the null hypothesis $(p_j^{(0)} = p_j^{(1)} = p_j)$, $\text{Cov}(\boldsymbol{y}_j, \boldsymbol{y}_j) = p_j(1-p_j) \cdot \boldsymbol{R}$. Hence

$$Var(\bar{y}_j^{(1)} - \bar{y}_j^{(0)}) = p_j(1-p_j)\boldsymbol{h}'\boldsymbol{R}\boldsymbol{h}.$$

To obtain $\hat{Var}(\bar{y}_j^{(1)} - \bar{y}_j^{(0)})$ for the test statistic, we need estimators $\hat{p}_j$ and $\hat{\boldsymbol{R}}$. It is straightforward that

$$\hat{p}_j = \bar{y}_j = \sum_{i=1}^{n} y_{ij}/n. \tag{1}$$

It is much more difficult to obtain $\hat{\boldsymbol{R}}$, which will be described in the following.

Based on the law of large numbers and the following two expressions (i' refers to the i'th isolate, $i \neq i'$)

$$\begin{aligned} E(y_{ij}y_{i'j}) &= E(y_{ij})E(y_{i'j}) + Cov(y_{ij}, y_{i'j}) \\ &= p_j^2 + \rho_{ii'}p_j(1-p_j) \end{aligned}$$

and

$$\mathrm{Var}(y_{ij}y_{i'j}) = E(y_{ij}y_{i'j})[1 - E(y_{ij}y_{i'j})],$$

$\sum_j y_{ij}y_{i'j}$ approximately follows a normal distribution:

$$N\left(\sum_j p_j^2 + \rho_{ii'}\sum_j p_j(1-p_j), \sum_j \left[p_j^2 + \rho_{ii'}p_j(1-p_j)\right]\left[1 - p_j^2 - \rho_{ii'}p_j(1-p_j)\right]\right). \quad (2)$$

Here $\rho_{ii'}$ is the correlation between the ith isolate and the i'th isolate, which is also the (i, i')th element of $\boldsymbol{R}$. By replacing p_j with $\hat{p}_j$, the MLE of $\rho_{ii'}$ can be solved from (2), the likelihood of $\sum_j y_{ij}y_{i'j}$.

However, we have observed that when the data is sparse, i.e., only a subset of isolates have observations at a particular position, the estimation of $\rho_{ii'}$ from the above procedure can be unstable. We propose to adopt a Bayesian approach. Instead of searching for the MLE from (2), we search for the MLE from the posterior distribution of $\rho_{ii'}$, which is proportional to the product of (2) and the prior distribution of $\rho_{ii'}$, denoted as $[\rho]$. Here $[\rho]$ is specified to reflect prior belief about plausible values of $\rho_{ii'}$, according to literature or expert opinion. One option is to specify $[\rho]$ as a beta distribution. The Bayesian approach effectively introduces a shrinkage in the estimates of $\rho_{ii'}$ toward their *a priori* plausible values, improving the stability of estimation.

Let $\hat{\boldsymbol{R}}$ be the estimated correlation matrix with elements being the estimated $\rho_{ii'}$. We can calculate

$$\hat{Var}(\bar{y}_j^{(1)} - \bar{y}_j^{(0)}) = \hat{p}_j(1 - \hat{p}_j)\boldsymbol{h}'\hat{\boldsymbol{R}}\boldsymbol{h}$$

and the test statistic

$$Z_j = \frac{\bar{y}_j^{(1)} - \bar{y}_j^{(0)}}{\sqrt{\hat{Var}(\bar{y}_j^{(1)} - \bar{y}_j^{(0)})}}.$$
